# Chimeric self-sufficient P450cam-RhFRed biocatalysts with broad substrate scope

**DOI:** 10.3762/bjoc.7.173

**Published:** 2011-11-02

**Authors:** Aélig Robin, Valentin Köhler, Alison Jones, Afruja Ali, Paul P Kelly, Elaine O'Reilly, Nicholas J Turner, Sabine L Flitsch

**Affiliations:** 1 School of Chemistry & Manchester Interdisciplinary Biocentre (MIB), University of Manchester, 131 Princess Street, Manchester M1 7DN, United Kingdom

**Keywords:** biocatalysis, C–H activation, high-throughput screening, P450 monooxygenase, substrate engineering

## Abstract

A high-throughput screening protocol for evaluating chimeric, self-sufficient P450 biocatalysts and their mutants against a panel of substrates was developed, leading to the identification of a number of novel biooxidation activities.

## Introduction

P450 monooxygenases are a ubiquitous family of enzymes found in a wide variety of organisms in all domains of life. These enzymes catalyse oxidation reactions such as hydroxylation, epoxidation, N- and O-dealkylation and heteroatom oxidation, often with high regio- and stereoselectivity [1], and are therefore attractive candidates for biocatalyst development. However, the need for reconstitution of protein redox partners, the necessary use of expensive NAD(P)H, and the lack of widely applicable high-throughput screening protocols render the development of efficient P450 biocatalysts a major challenge. In recent years, naturally fused P450 enzymes, such as P450BM-3 [2–7] and P450RhF [8–11], have attracted great interest, although they represent only a small fraction of the thousands of naturally occurring P450 enzymes in nature. We and others have recently reported highly active chimeric P450s in which the P450 domain of a variety of bacterial enzymes is fused with the reductase domain of the self-sufficient P450RhF [12–14].

**Figure 1:**
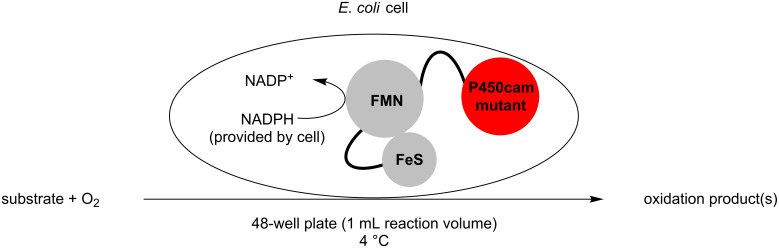
Oxidation reactions with P450cam-RhFRed mutants.

Given that these chimeric enzymes can function in bacterial hosts, we proposed that such whole-cell P450 systems would be amenable to incorporation into a high-throughput screening protocol in multiwell plates. Figure 1 outlines the design of the screening platform: *E.coli* hosts containing a variety of easily engineered chimeric constructs are incubated with the substrate under physiological conditions, and the oxidation products are then analysed by standard analytical methods, such as GC–MS.

## Results and Discussion

**Scheme 1:**
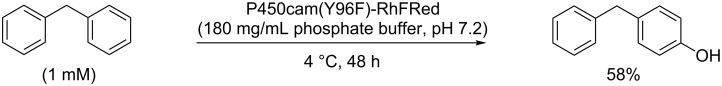
Whole cell biotransformation of diphenylmethane to 4-hydroxydiphenylmethane with P450cam(Y96A)-RhFRed.

Our previous work showed that the chimera P450cam-RhFRed allows 100% conversion of camphor to 5-*exo*-hydroxycamphor at 3 mM substrate concentration. The substrate specificity of P450cam can be significantly broadened by specific mutations, in particular by mutating the tyrosine residue at position 96 (Y96) in combination with other sites such as V247 [15–22]. Exchanging Y96 for an alanine residue allows the oxidation of unnatural substrates, presumably because it opens up the active site. S. Flitsch, L.-L Wong and coworkers for example showed that P450cam (Y96A) was able to hydroxylate diphenylmethane to 4-hydroxydiphenylmethane, an activity that was not observed with the wild-type enzyme [15]. For the present study, incorporation of the Y96A mutation into our P450cam-RhFRed fused system made the whole cell biotransformation of diphenylmethane as a substrate possible (4 mL reaction in a 15 mL Falcon tube) with a GC–MS yield of 58% of 4-hydroxydiphenylmethane after 48 h (Scheme 1).

In order to improve screening throughput, we investigated the whole cell biotransformation of diphenylmethane with P450cam(Y96A)-RhFRed in multiwell plates (24, 48 and 96) with different reaction volumes (0.5 mL, 1 mL and 2 mL). The biotransformation carried out in a 48-well plate with a reaction volume of 1 mL was found to give a similar conversion to the 4 mL reaction in a Falcon tube, and this format was therefore adopted for subsequent screening of P450cam-RhFRed variants. In addition to Y96A, mutations Y96F and Y96F/V247A were incorporated into the P450cam-RhFRed system for investigation, having shown interesting broad substrate ranges in previous studies with nonfused P450cam.

Initially, the microtitre-plate system was optimised and shown to give similar yields to our previously reported biotransformations. The three fused P450cam-RhFRed variants were screened against a panel of substrates, which were selected on the basis of the potential application of their oxidation products. For instance, the terpenes α- and β-ionone and their derivatives have been widely used for the synthesis of carotenoids or in the fragrance industry [23–26], and functionalized aliphatic heterocycles are versatile building blocks for further elaboration, e.g., by biocatalytic means [27].

The three P450cam-RhFRed mutants were expressed in *E. coli* as previously described [12–14] and their presence confirmed by SDS–PAGE (see Supporting Information File 1). The screening was carried out at 4 °C in 48-well plates with a reaction volume of 1 mL (180 mg wet cells/mL phosphate buffer, pH 7.2) and a substrate concentration of 1 mM. After 24 h and 48 h aliquots of the reaction mixture were extracted with ethyl acetate and analysed by GC–MS. In the screening process, previously undescribed biocatalytic activities were discovered (Table 1). Thus, the monoterpenes **1**, **3** and **5** were hydroxylated by all three P450cam-RhFRed mutants, with the double mutant Y96F/V247A showing >99% conversion after 24 h (only one hydroxylated product was detected in the extracts).

**Table 1:** Conversion (% relative peak-area by GC–MS of extracted reaction mixtures), after 24 h, of substrates **1**, **3**, **5**, and, after 48 h, of substrates **7**, **11**, **13** and **15** with P450cam-RhFRed mutants in 48-well plate biotransformations.

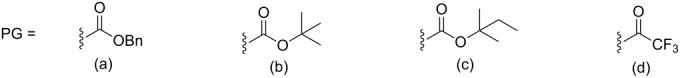						

Compounds				P450cam-RhFRed mutants		
		
Substrate	Products	Substrate no.		Y96A	Y96F	Y96F/V247A

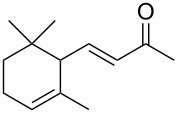 **1**	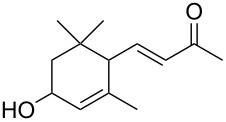 **2**	**1**		>99	>99	>99

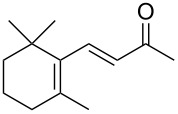 **3**	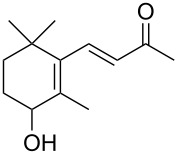 **4**	**3**		94	98	>99

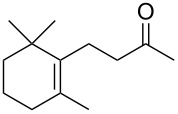 **5**	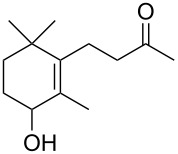 **6**	**5**		64	50	>99

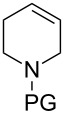 **7**	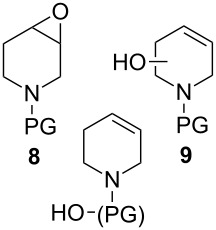 **10**	**7a**		12 (**8a**)	—	—

		**7b**		48 (**8b**)	26 (**8b**)	73 (**8b**)^a^

		**7c**		85 (**10c**)^b^	16 (**8c**) 34 (**10c**)	7 (**8c**) 59 (**10c**) 31 (**10c'**)

		**7d**		n.c.^c^	n.c.^c^	n.c.^c^

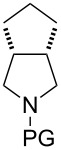 **11**	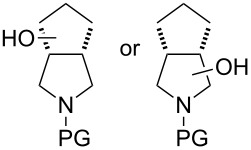 **12**	**11a**		<2 (u)^d^	—	—

		**11b**		12 (**12b**)	<2 (u)^d^	9 (**12b**) 15 (**12b'**)

		**11c**		<2 (u)^e^	n.c.^c^	11 (**12c**)

		**11d**		88 (**12d**)^f^	22 (**12d**) 69 (u)^d^	18 (**12d**) 82 (u)^d^

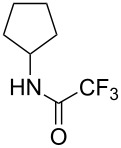 **13**	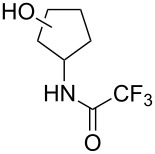 **14**	**13**		n.c.^c^	n.c.^c^	18 (**14d**) 6 (**14d'**)

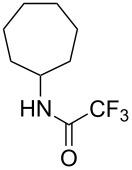 **15**	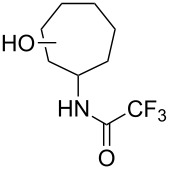 **16**	**15**		15 (**16d**) 9 (**16d'**)	11 (**16d**) 10 (**16d'**)	15 (**16d**) 75 (**16d'**)

^a^One other product was detected (8%). ^b^One other product was detected (10%). ^c^n.c. = no conversion. ^d^u = unidentified product. ^e^Two unidentified products. ^f^Three other minor products were detected (12%).

Epoxidation of tetrahydropyridine **7a** to compound **8a** with P450cam(Y96A)-RhFRed was shown to occur with low conversion (12%) with carboxybenzyl as a protecting group. In order to optimise the epoxidation reaction further, the N-protection group was used as another variable in the screening and a variety of N-protecting groups were installed to generate a set of tetrahydropyridine derivatives **7**. An increase in conversion (48%) was obtained with *tert*-butyloxycarbonyl (Boc, compound **7b**) instead of benzyloxycarbonyl (Cbz, compound **7a**) protection. In some product mixtures from **7b** additional peaks of hydroxylated compounds were observed, which could correspond to structures **9**, but these were not further characterised. Surprisingly, compound **7c**, bearing a protecting group very similar to Boc, showed mainly hydroxylation of an unactivated carbon atom of the protecting group. Mutant P450cam(Y96F)-RhFRed displayed similar results to the Y96A mutant, albeit with lower conversions. The double mutant P450cam(Y96F/V247A)-RhFRed turned out to be the most efficient for the epoxidation of the tetrahydropyridine ring, with substrate **7b** epoxidised to **8b** in 73% conversion. Racemic epoxide **8b** was synthesised to confirm the structure of the biotransformation product and to determine the enantiomeric excess. This was found to be 18% for the biotransformation reaction with P450cam(Y96A)-RhFRed, 13% with P450cam(Y96F)-RhFRed and 15% with P450cam(Y96F/V247A)-RhFRed.

Similar studies were performed with compound **11** in order to find the combination of P450 mutant and protecting group with the highest hydroxylation conversion. Low conversions or mixtures of two products were obtained with compounds **11a**, **11b** and **11c**. Interestingly, when the trifluoroacetyl protecting group was used (**11d**) no starting material was detected in the EtOAc extract of the reaction mixture after 48 h. The mutant P450cam(Y96A)-RhFRed was shown to convert **11d** to one major hydroxylated product (**12d**, 88%), with three minor unidentified products also detected (12%). The other two mutants also showed high hydroxylation activity with trifluoroacetyl-protected **11**.

Similar results were obtained with cyclopentylamine and cycloheptylamine derivatives **13** and **15**. In the case of compound **13**, only the mutant P450cam(Y96F/V247A) was able to hydroxylate the ring, and this gave a mixture of two hydroxylated products. Compound **15** was also shown to give two hydroxylated products, again with the mutant P450cam(Y96F/V247A) showing the highest conversions. The regioselectivity of the hydroxylation of compounds **11**, **13** and **15** has not yet been determined and is currently under investigation.

## Conclusion

In summary, we have described a fast screening method that allows us to find new biocatalysts for oxidation reactions, either by screening P450 variants or by screening substrate variants through variation of the protecting group. The combination of these reaction parameters allowed for the optimisation of a specific biocatalytic reaction. The biocatalysts are very easy to generate as bacterial whole-cell systems and the screening protocol should make this method attractive for the further discovery and development of new oxidation activity.

## Supporting Information

The Supporting Information features the full experimental procedures, characterisation of the synthesised, previously unknown compounds, GC–MS traces of the biotransformation reactions and the MS traces of the corresponding starting materials.

File 1 Experimental part and GC–MS traces.
