# Supplementary material for: Chimeric self-sufficient P450cam-RhFRed biocatalysts with broad substrate scope
Source: Beilstein J Org Chem. 2011 Nov 2;7:1494–8. doi: 10.3762/bjoc.7.173 (PMC3252848; doi:10.3762/bjoc.7.173)
Supplement: File 1 — Experimental part and GC–MS traces. [file Beilstein_J_Org_Chem-07-1494-s001.pdf]

Supporting Information

for

Chimeric self-sufficient P450cam-RhFRed biocatalysts with broad substrate scope

Aélig Robin, Valentin Köhler, Alison Jones, Afruja Ali, Paul P. Kelly, Elaine O'Reilly, Nicholas J. Turner and Sabine L. Flitsch*

Address: School of Chemistry & Manchester Interdisciplinary Biocentre (MIB), University of Manchester, 131 Princess Street, Manchester M1 7DN, United Kingdom

E-mail: Sabine L. Flitsch* - sabine.flitsch@manchester.ac.uk

*Corresponding author

Experimental part and GC–MS traces.

Chemicals and Bacterial Strains S2

Synthesis of starting materials S3

Synthesis of Cbz-protected amines S3

Synthesis of Boc-protected amines S4

Protection of amines as tert-amyl carbamates S5

Protection of amines as trifluoroacetamides S6

Engineering of P450cam(Y96A)-RhFRed S8
Engineering of P450cam-RhFRed mutants Y96F and Y96F/V247A S8
Production of P450cam-RhFRed mutants (Y96A, Y96F and Y96F/V247A).......... S9
Whole cell biotransformations in 48-well plates (1 mM substrate concentration) S10
GC analysis S10
GC–MS traces.......... S11
*Biotransformation of α-ionone **1** (1 mM) with P450cam(Y96F/V247A)-RhFRed* S11
*Biotransformation of β-ionone **3** (1 mM) with P450cam(Y96F/V247A)-RhFRed*.......... S13
*Biotransformation of dihydro-β-ionone **5** (1 mM) with P450cam(Y96F/V247A)-* S15
*Biotransformation of compound **7b** (1 mM) with P450cam(Y96F/V247A)-RhFRed* S17
*Biotransformation of compound **7c** (1 mM) with P450cam(Y96F)-RhFRed* S19
*Biotransformation of compound **11** (1 mM) with P450cam(Y96A)-RhFRed* S22
*Biotransformation of compound **13** (1 mM) with P450cam(Y96F/V247A)-RhFRed* S23
*Biotransformation of compound **15** (1 mM) with P450cam(Y96F/V247A)-RhFRed* S26
References. S28

Chemicals and Bacterial Strains 3-Azabicylo[3.3.0]octane hydrochloride was purchased from AK Scientific. All other chemicals were purchased from Sigma-Aldrich, Fisher Scientific and TCI Europe. *Escherichia coli* XL1 Blue supercompetent cells were obtained from Stratagene (La Jolla, CA) and *E. coli* BL21(DE3) from Invitrogen (Carlsbad, CA). Solvents were of analytical or HPLC grade or were purchased as dried over molecular sieves, where necessary. Column chromatography was performed on silica gel (Fluka, 220–440 mesh). Petrol ether, used for column chromatography, had a boiling point between 40 °C and 60 °C. ^{1}H and ^{13}C NMR spectra were recorded on a Bruker Avance 400 spectrometer (400.1 MHz for ^{1}H and 100.6 MHz for ^{13}C) in $CDCl_3$ without an additional standard. Chemical shifts are reported in δ values (ppm) and are relative to the solvent signal ($CHCl_3$, ^{1}H = 7.26; $CDCl_3$, ^{13}C = 77.0). Elemental analyses were obtained from the Chemistry Department of the University of Manchester. Mass

spectra were recorded on a Waters Platform II instrument. GC–MS spectra were recorded on a Hewlett Packard HP 6890 equipped with a HP-1MS column, a HP 5973 Mass Selective Detector and an ATLAS GL FOCUS sampling robot. GC analysis was performed on Agilent 6850 GCs equipped with a Gerstel MultiPurpose Sampler MPS2L and a Varian CP-Chirasil-DEX CB column or an HP-1 column.

Synthesis of starting materials

Synthesis of Cbz-protected amines

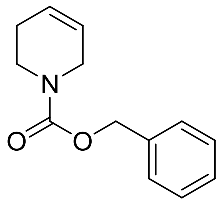

7a

For a comparison with literature NMR data see, e.g., [1]. 1,2,3,6-tetrahydropyridine (1.10 mL, 12 mmol, 1.2 equiv) was stirred in 3 N aqueous NaOH solution (3.67 mL, 1.1 equiv) and the mixture cooled to 0 ºC. Benzyl chloroformate (1.41 mL, 10.0 mmol, 1.0 equiv) was added dropwise and the mixture allowed to warm to rt and stirred for 3 h. Subsequently the mixture was extracted with *tert*-butyl methyl ether (2 × 50 mL). The combined organic extracts were washed with 0.1 N aqueous HCl (3 × 100 mL) followed by saturated aqueous $NaHCO_3$ solution (1 × 50 mL). The crude product (1.968 g) was purified by column chromatography over silica (petrol ether/EtOAc 5:1, R_f 0.18). Colorless oil (23% yield). ^{1}H NMR (400.1 MHz, $CDCl_3$, 294 K) δ 7.43–7.27 (m, 5H), 5.82 (m, 1H), 5.74–5.54 (m, 1H), 5.16 (‘bs’, 2H), 3.96 (m, 2H), 3.57 (m, 2H), 2.14 (m, 2H); ^{13}C NMR (100.6 MHz, $CDCl_3$, 294 K; mixture of rotamers; only signals for major rotamer listed; signals assigned according to signal height) δ 155.4, 136.7, 128.3, 127.8,

127.7, 125.3, 123.7, 66.8, 43.2, 40.0, 24.7; MS (ESI+) *m/z* 489 ([2M + MeOH + Na^+], 21), 485 (26), 457 ([2M + Na^+], 25), 318 (10), 240 ([M + Na^+], 100).

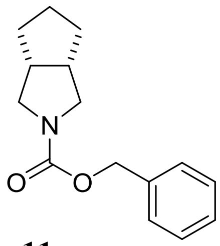

11a

3-Azabicyclo[3.3.0]octane hydrochloride (1.77 g, 12 mmol, 1.2 equiv) was treated with 3 N aqueous NaOH solution (7.67 mL, 2.3 equiv) and benzyl chloroformate (1.41 mL, 10.0 mmol, 1.0 equiv) followed by workup as described for the preparation of Cbz-protected 1,2,3,6-tetrahydropyridine with the difference that the reaction mixture was stirred overnight. The crude product was analytically pure and used as obtained. Colorless oil (2.36 g, 96% yield). ^{1}H NMR (400.1 MHz, $CDCl_3$, 295 K) δ 7.40–7.25 (m, 5H), 5.12 (s, 2H), 3.60 (dd, *J* = 11.3, 8.1 Hz, 2H), 3.19 (dd, *J* = 11.3, 4.0 Hz, 2H), 2.62 (m, 2H), 1.86–1.67 (m, 3H), 1.66–1.51 (m, 1H), 1.50–1.37 (m, 2H); ^{13}C NMR (100.6 MHz, $CDCl_3$, 295 K) δ 154.8, 137.1, 128.4, 127.9, 127.8, 66.1, 52.2, 42.9 (b), 32.0, 25.6; MS (ESI+) *m/z* 513 ([2M + Na^+], 60), 346 (23), 300 ([M + MeOH + Na^+], 30), 268 ([M + Na^+], 100), 246 ([M + H^+], 26); Elem. Anal.: calcd. for $C_{15}H_{19}NO_2$: C, 73.44; H, 7.81; N, 5.71; found C, 73.09; H, 8.10; N, 5.63.

Synthesis of Boc-protected amines

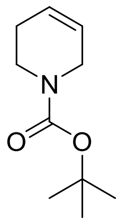

7b

For comparison with literature ^{1}H NMR data see, e.g., [2]. A solution of Boc_2O (2.30 mL, 10.0 mmol, 1.0 equiv) in dry THF (10 mL) was cooled to 0 ºC in an ice bath. 1,2,3,6-Tetrahydropyridine (1.37 mL, 15.0 mmol, 1.5 equiv) was added dropwise and the solution was allowed to warm to rt and stirred overnight. Subsequently saturated aqueous $NaHCO_3$ solution (10 mL) was added and the

mixture extracted with *tert*-butyl methyl ether (3 × 25 mL). The combined organic extracts were washed with 0.1 N aqueous HCl (3 × 100 mL) followed by saturated aqueous $NaHCO_3$ solution (1 × 50 mL). The organic phase was dried with $MgSO_4$ and the volatiles removed by means of a rotary evaporator. Colorless oil (1.63 g, 89% yield). ^1H NMR (400.1 MHz, $CDCl_3$, 295 K) δ 5.83–5.74 (m, 1H), 5.67–5.57 (m, 1H), 3.84 ('dt', *J* = 5.3, 2.9 Hz, 2H), 3.45 ('t', *J* = 5.8 Hz, 2H), 2.09 (m, 2 H), 1.44 (s, 9H); ^{13}C NMR (100.6 MHz, $CDCl_3$, 295 K) δ 154.9, 125.1, 124.4, 79.3, 43.3, 40.0 (b), 28.4, 25.1; MS (ESI+) *m/z* 389 ([2M + Na^+], 33), 238 ([M + MeOH + Na^+], 56), 206 ([M + Na^+], 100).

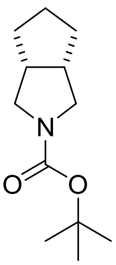

11b

The compound was prepared in analogy to Boc-protected 1,2,3,6-tetrahydropyridine starting from 3-azabicylo[3.3.0]octane (1.00 g, 8.99 mmol). Colorless oil (93% yield). ^1H NMR (400.1 MHz, $CDCl_3$, 294 K) δ 3.47 (dd, *J* = 11.2, 8.3 Hz, 2H), 3.04 (dd, *J* = 11.2, 4.1 Hz, 2H), 2.55 (m, 2H), 1.83–1.61 (m, 3H), 1.61–1.47 (m, 1H), ~1.46–1.33 (m, overlapped by a signal at 1.40 ppm, 2H) 1.40 (s, 9H); ^{13}C NMR (100.6 MHz, $CDCl_3$, 294 K) δ 154.5, 78.8, 51.9, 42.8, 31.9, 28.4, 25.4; MS (ESI+) *m/z* 266 ([M + MeOH + Na^+], 32), 234 ([M + Na^+], 100).

Protection of amines as tert-amyl carbamates

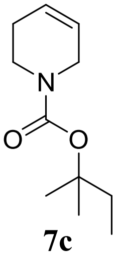

7c

The protection and work up was carried out as described for Boc-protection. Light-yellow oil (80% yield). ^1H NMR (400.1 MHz, $CDCl_3$, 294 K) δ 5.84–5.69 (m, 1H), 5.67–5.50 (m, 1H), 3.90–3.75 (m, 2H), 3.51–3.34 (m, 2H), 2.07

(m, 2H), 1.73 (‘q’, J = 7.5 Hz, 2H), 1.39 (‘s’, 6H), 0.84 (‘t’, J = 7.5 Hz, 3H); ^{13}C NMR (100.6 MHz, $CDCl_3$, 294 K) δ 154.8, 125.1, 124.4 (b), 81.6, 43.2, 40.0 (b), 33.8, 25.8, 25.0, 8.2; MS (ESI+) *m/z* 463 (21), 449 ([2M + 2MeOH + Na^+], 15), 417 ([2M + Na^+], 25), 252 ([M + MeOH + Na^+], 83), 220 ([M + Na^+], 100).

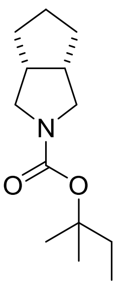

11c

The crude product was purified by column chromatography (petrol ether/EtOAc 10:1) over silica. Colorless liquid (75% yield). ^{1}H NMR (400.1 MHz, $CDCl_3$, 294 K) δ 3.57–3.46 (m, 2H), 3.14–3.05 (m, 2H), 2.65–2.54 (m, 2H), 1.84–1.69 (m, 3H), 1.75 (q, J = 7.5 Hz, 2H), 1.62–1.51 (m, 1H), 1.48~1.36 (m, 2H), 1.42 (bs, 6H), 0.89 (t, J = 7.5 Hz, 3H); ^{13}C NMR (100.6 MHz, $CDCl_3$, 295 K) δ 154.5, 81.2, 52.0, 42.8, 34.0, 32.0, 25.9, 25.5, 8.3, MS (ESI+) *m/z* 526 (18), 473 ([2M + Na^+], 100), 451 ([2M + H^+], 44), 299 (26), 280 ([M + MeOH + Na^+], 87), 269 (40), 248 ([M + Na^+], 75), 226 ([M + H^+], 27).

Protection of amines as trifluoroacetamides

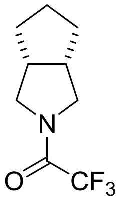

11d

A solution of 3-azabicylo[3.3.0]octane (556 mg, 5.00 mmol) in dry CH_2Cl_2 (10 mL) was cooled in an ice bath and trifluoroacetic anhydride (0.695 mL, 1.05 g, 5.00 mmol) was added dropwise. After warming to rt the mixture was stirred overnight. For workup the mixture was diluted with CH_2Cl_2 (10 mL) and washed with 0.1 N aqueous hydrochloric acid (3 x 20 mL) and sat. aq. $NaHCO_3$ solution (3 x 20 mL). The product was obtained as a colorless liquid (61% yield). ^{1}H NMR (400.1 MHz, $CDCl_3$, 294 K) δ 3.80–3.66 (m, 2H), 3.43–3.33 (m, 2H), 2.79–2.59 (m, 2H), 1.92–1.78 (m, 2H), 1.78–1.55 (m, 2H), 1.52–1.35 (m, 2H); ^{13}C NMR

(100.6 MHz, $CDCl_3$, 295 K, hindered rotation around C(O)–N bond, signals listed as observed) δ 155.1 (q, *J* = 36.6 Hz), 116.3 (q, *J* = 287.3 Hz), 53.0, 52.2 (q, *J* = 3.1 Hz), 43.7, 40.9, 31.9, 31.5, 25.3; MS (ESI+) *m/z* 473 ([M + MeOH + Na^+], 40), 230 ([M + Na^+], 100), 208 ([M + H^+], 13).

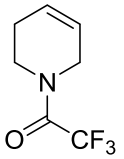
7d

Almost colorless oil. ^1H NMR (400.1 MHz, $CDCl_3$, 294 K, mixture of rotamers) δ 5.93–5.75 (m, 1H), 5.72–5.57 (m, 1H), 4.11–3.98 (m, 2H), 3.76–3.55 (m, 2H), 2.22 (m, 2H); ^{13}C NMR (100.6 MHz, $CDCl_3$, 294 K, mixture of rotamers - only signals for major rotamer listed; signals assigned according to signal height) δ 155.7 (q, *J* = 35.7 Hz), 125.0, 123.1, 116.4 (q, *J* =287.8 Hz), 43.1, 42.6 (q, *J* = 3.5 Hz), 25.5; MS (ESI+) *m/z* 381 ([2M + Na^+], 7), 234 ([M + MeOH + Na^+], 46), 202 ([M + Na^+], 100).

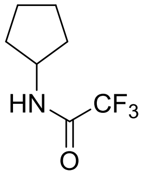
13

The crude product was recrystallized from hot pentane upon cooling to rt. Beige solid (38% yield). ^1H NMR (400.1 MHz, $CDCl_3$, 294 K) δ 6.29 (bs, 1H), 4.23 ('sext', *J* = 7 Hz, 1H), 2.12–1.96 (m, 2H), 1.80–1.57 (m, 4H), 1.54–1.41 (m, 2H); ^{13}C NMR (100.6 MHz, $CDCl_3$, 295 K) δ 156.7 (q, *J* = 36.6 Hz), 115.8 (q, *J* = 288.3 Hz), 51.8, 32.6, 23.6; MS (ESI+) *m/z* 236 ([M + MeOH + Na^+], 19); 204 ([M + Na^+], 100); Elem. Anal.: calcd. for $C_7H_{10}F_3NO$: C, 46.41; H, 5.56; N, 7.73; found C, 46.32; H, 5.53; N, 7.65.

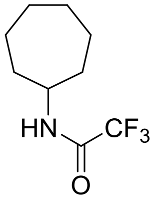
15

Beige, microcrystalline solid (71% yield). ^1H NMR (400.1 MHz, $CDCl_3$, 294 K) δ 6.21 (bs, 1H), 3.98 (m, 1H), 2.06–1.88 (m, 2H), 1.73–1.41 (m,

10H); ^{13}C NMR (100.6 MHz, $CDCl_3$, 295 K) δ 156.0 (q, J = 36.7 Hz), 115.9 (q, J = 287.1 Hz), 51.4, 34.4, 27.8, 23.8; MS (ESI+) *m/z* 264 ([M + MeOH + Na^+], 38), 232 ([M + Na^+], 100); Elem. Anal.: calcd. for $C_9H_{14}F_3NO$: C, 51.67; H, 6.75; N, 6.70; found C, 51.91; H, 6.82; N, 6.69.

Engineering of P450cam(Y96A)-RhFRed As previously described [3].

Engineering of P450cam-RhFRed mutants Y96F and Y96F/V247A We performed PCR to mutate residues Y96 and V247 in the P450cam active site. The sequences of the oligonucleotides used as primers for the PCR are listed in Table 1. The nucleotide exchanges are underlined. The reactions were carried out as described in the QuikChange® site-directed mutagenesis protocol by using the following thermal profile cycle (16 cycles): 95 °C for 30 s/55 °C for 1 min/68 °C for 14 min.

	PCR primers<br>*(DNA template, mass DNA template, annealing temperature)*
P450cam(Y96F)-RhFRed	forward 5′-CTCGTGAAGCCGGCGAAGCCTTTGACTTCATTCCCACCTCGATGG-3′<br>reverse 5′-CCATCGAGGTGGGAATGAAGTCAAAGGCTTCGCCGGCTTCACGAG-3′<br>*(P450cam(Y96A)-RhFRed, 50 ng)*
P450cam(Y96F/V247A)-RhFRed	forward 5′-GAGGATGTGTGGCCTGTTACTGGCAGGCGGCCCTGGATACG-3′<br>reverse 5′-CGTATCCAGGGCCGCCTGCCAGTAACAGGCCACACATCCTC-3′<br>*(P450cam(Y96F)-RhFRed, 50 ng)*

Table 1: PCR conditions and primers for the engineering of P450cam-RhFRed mutants Y96F and Y96F/V247A.

Production of P450cam-RhFRed mutants (Y96A, Y96F and Y96F/V247A)

A single colony of *E. coli* BL21(DE3) cells containing plasmid P450cam(Y96A)-RhFRed or P450cam(Y96F)-RhFRed or P450cam(Y96F/V247A)-RhFRed was picked from overnight plates and used to inoculate 5 mL LB medium supplemented with 100 µg of ampicillin/mL. The culture was grown overnight at 37 °C in a shaking incubator (250 rpm). Cells were harvested by centrifugation, resuspended in culture medium and used to inoculate 500 mL of M9 medium [4] containing 100 µg ampicillin/mL, 0.4% of glucose and 0.05% of $FeCl_3$ in a 2 L flask. Cells were grown at 37 °C to an optical density (OD_{600}) of 0.8. Following induction with β-D-thiogalactopyranoside (IPTG) at 0.4 mM and addition of 5-aminolevulinic acid hydrochloride (ALA) at 0.5 mM, the cells were grown for a further 16 h at 25 °C ($2.2 < OD_{600} < 2.8$). Expression of P450cam-RhFRed mutants was confirmed by analysing cell extracts with SDS–polyacrylamide gel electrophoresis (SDS–PAGE). Maximum enzyme activity was maintained by repeated subcloning of the fusion gene into pET14b rather than propagation of expression vector DNA stocks.

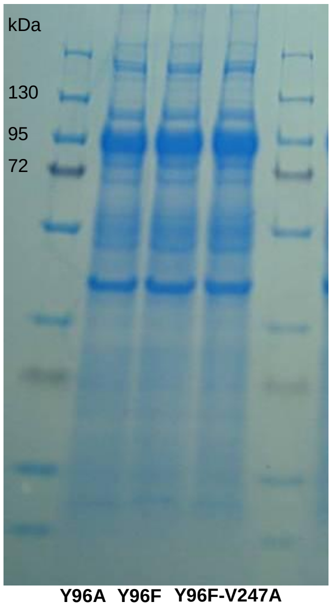

Figure 1: SDS–PAGE analysis of the soluble fractions showing the expression of P450cam-RhFRed mutants in *E. coli*. The calculated molecular mass of the proteins is about 81 kDa.

Whole cell biotransformations in 48-well plates (1 mM substrate concentration) After overnight growth, the culture was centrifuged (8000 rpm, 20 min, 4 °C) and the wet cells (2.5 g) were resuspended in 50 mM phosphate buffer (pH 7.2, 13.9 mL, 180 mg wet cells/mL of phosphate buffer) containing 0.4% of glycerol (added for the stabilization of the enzyme). 1 mL of resuspended cell mixture was then transferred into one well of a 48-well plate and the substrate was added (26.6 μL of a 37.5 mM substrate solution in DMSO). The plate was subsequently placed in a shaking incubator (250 rpm) for 48 h at 4 °C. 200 μL aliquots of the reaction mixture were collected after 48 h and extracted with 500 μL of ethyl acetate. The organic phase was analysed by GC–MS or GC–FID.

GC analysis Analysis was performed on a Hewlett Packard GC–MS (HP 6890 series/HP 5973) or on an Agilent 6850 GC–FID. Helium was used as the carrier gas at a flow rate of 1.6 mL/min. GC–FID was equipped with an Agilent HP-1 column (30 m, 0.32 mm i.d, 0.25 μm film thickness) or CP-Chirasil-DEX CB (24.8 m, 0.25 mm i.d, 0.26 μm film thickness), GC–MS with an Agilent HP-1MS column of the same dimensions. The temperature programs are summarised in Table 2. The temperatures were 270 °C for the injection and 250 °C for the detector (GC–FID) (250 °C for the injection and 275 °C for the detector for chiral GC analysis). Sample volume was 1 μL and samples were injected at a split ratio of 10:1 (100:1 for chiral GC).

	Oven temperatures				
Substrates	Initial Temperature (hold time)	Gradient 1 (°C / min)	Temperature 1 (hold time)	Gradient 2 (°C / min)	Temperature 2 (hold time)
Ionones **1**, **3**, **5**	100 °C (1 min)	4	220 °C	30	250 °C
**7a–c**, **11a–c**	110 °C	15	250 °C (5 min)	20	300 °C (2 min)
**7d**, **11d**, **13**, **15**	70 °C	15	250 °C (5 min)	20	300 °C (2 min)
chiral analysis **8b**	130 °C (30 min)	15	190 °C (5 min)	–	–

Table 2: Temperature programs for GC analysis of substrates **1**, **3**, **5**, **7a–d**, **11a–d**, **13**, **15** and their oxidative product(s) and chiral GC analysis for **8b** (CP-Chirasil-DEX CB column).

GC–MS traces

Biotransformation of α-ionone ***1*** *(1 mM) with P450cam(Y96F/V247A)-RhFRed*

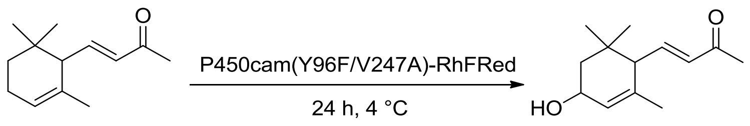

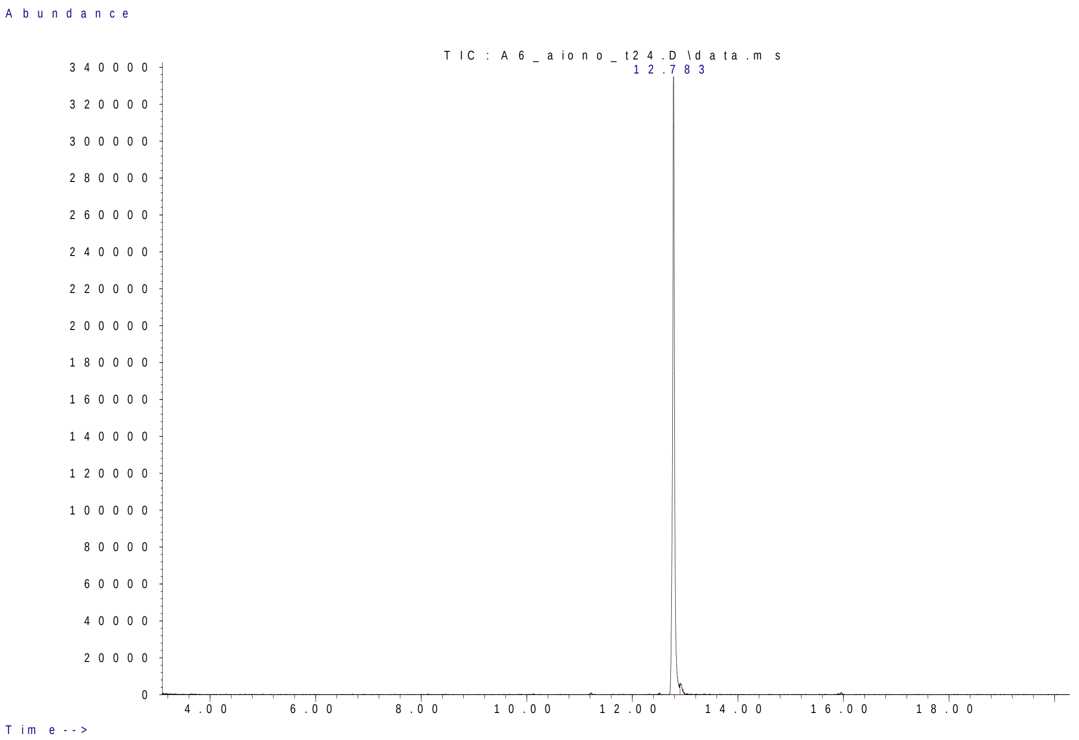

α-ionone **1** (*m/z* = 192): 8.120 min

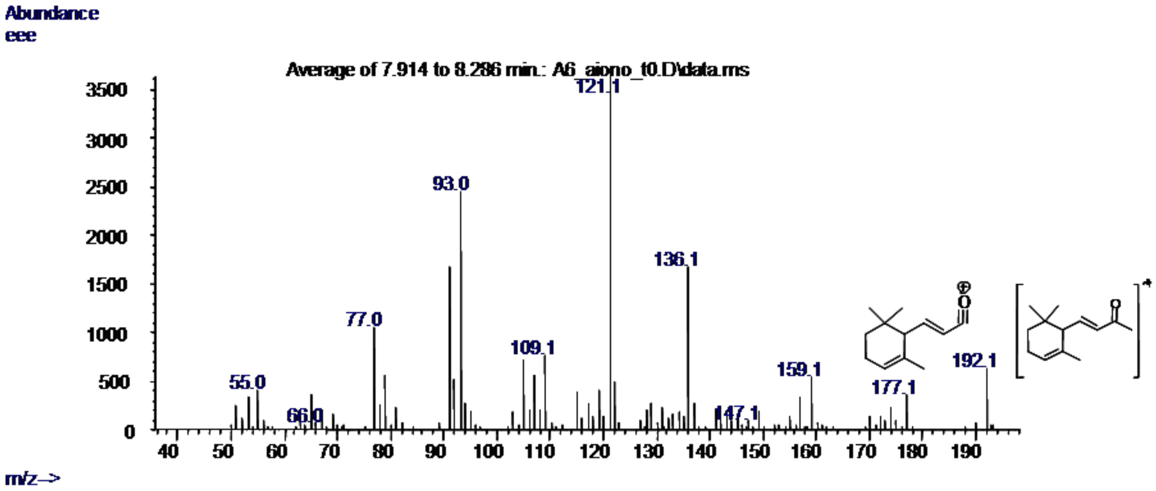

3-Hydroxy-α-ionone (m/z = 208): 12.783 min. Assignment of hydroxylation regioselectivity was based on NMR studies and by comparison with literature MS-data [5].

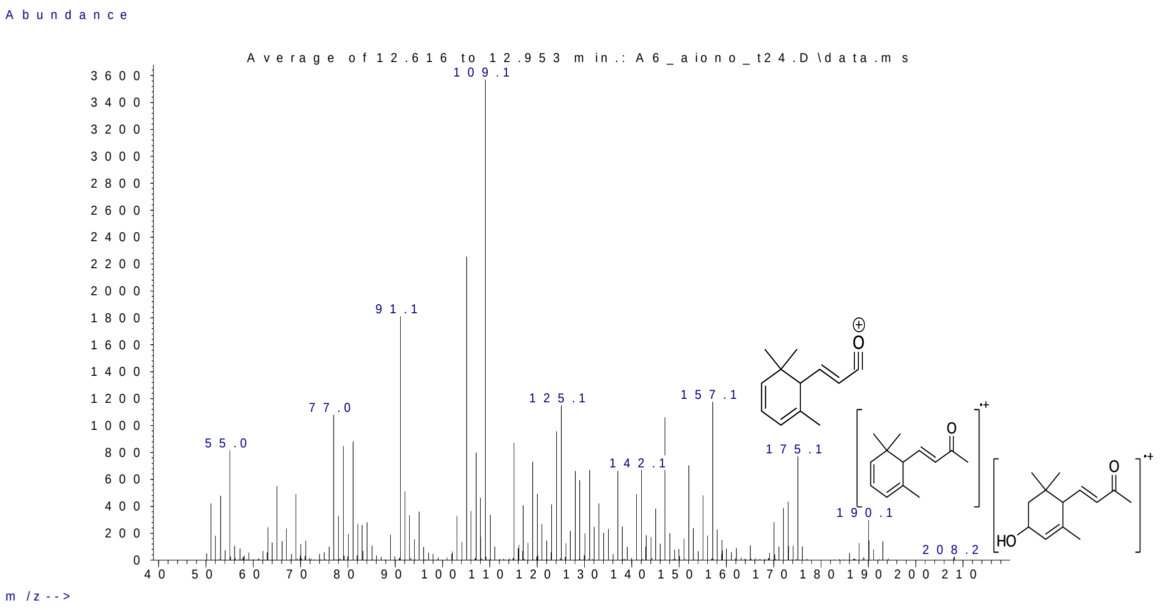

Biotransformation of β-ionone ***3*** *(1 mM) with P450cam(Y96F/V247A)-RhFRed*

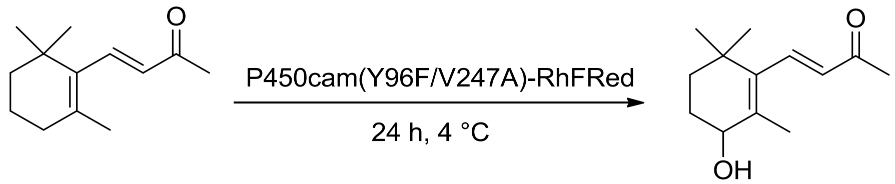

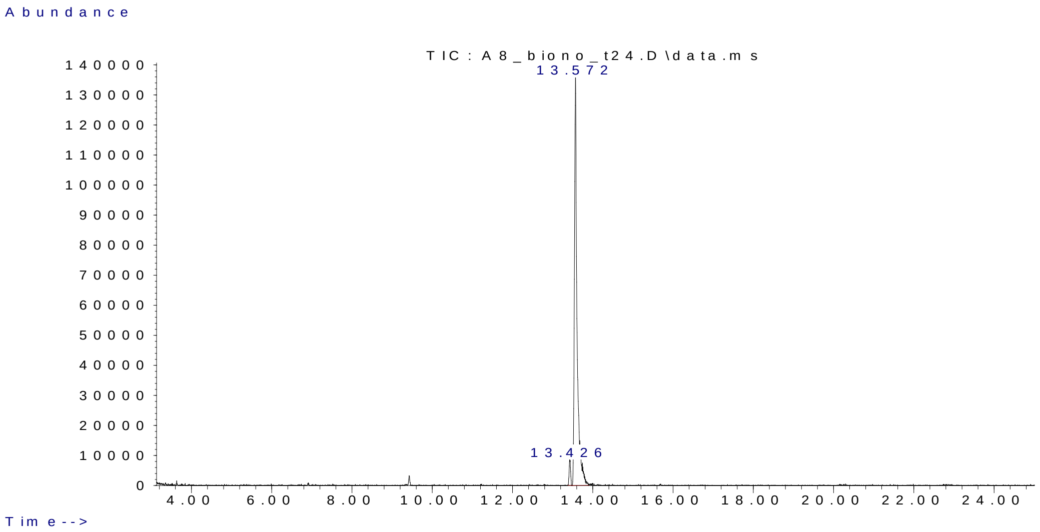

β-ionone **3** (*m/z* = 192): 9.433 min

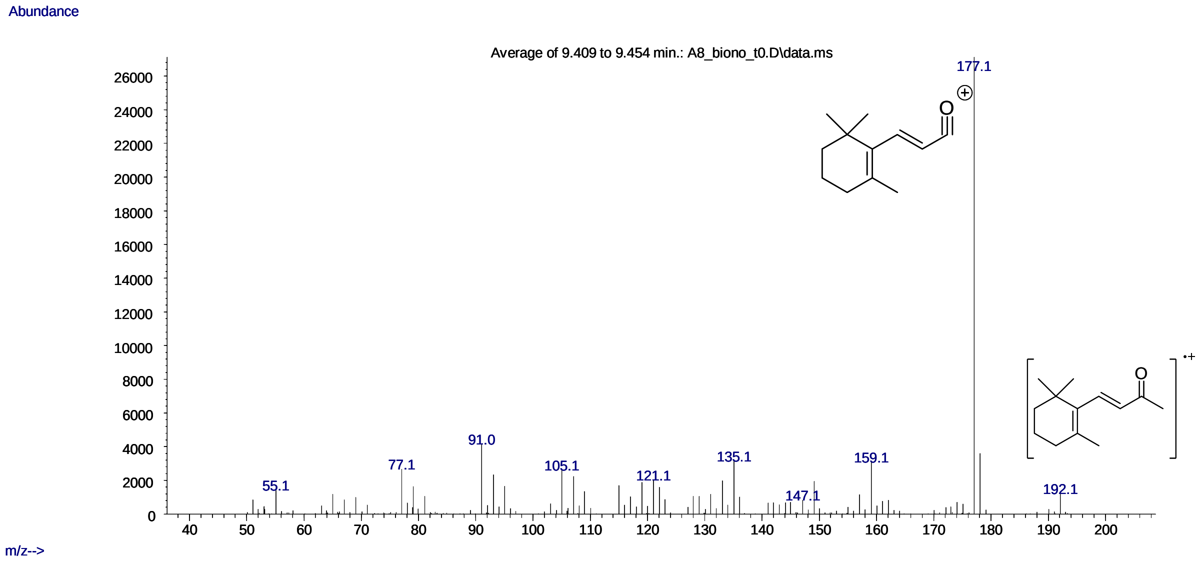

4-hydroxy-β-ionone (*m/z* =208): 13.572 min. Assignment of hydroxylation regioselectivity was based on NMR studies and by comparison with literature MS-data [6].

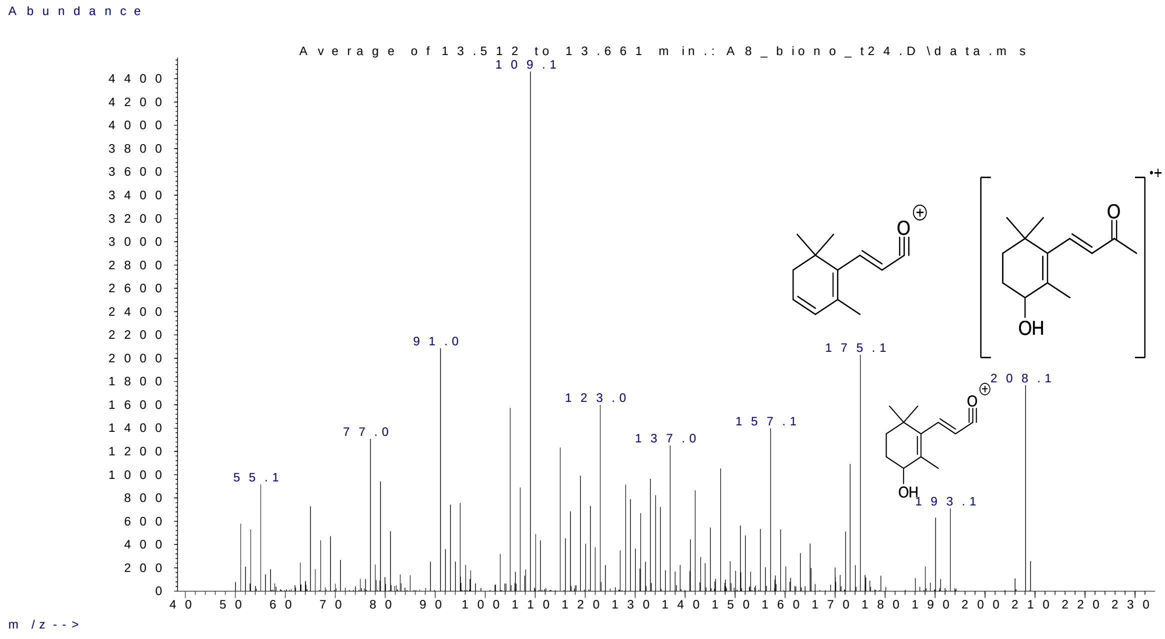

*Biotransformation of dihydro-β-ionone **5** (1 mM) with P450cam(Y96F/V247A)-RhFRed*

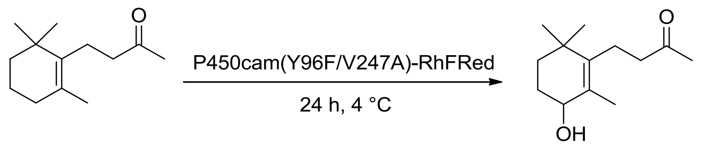

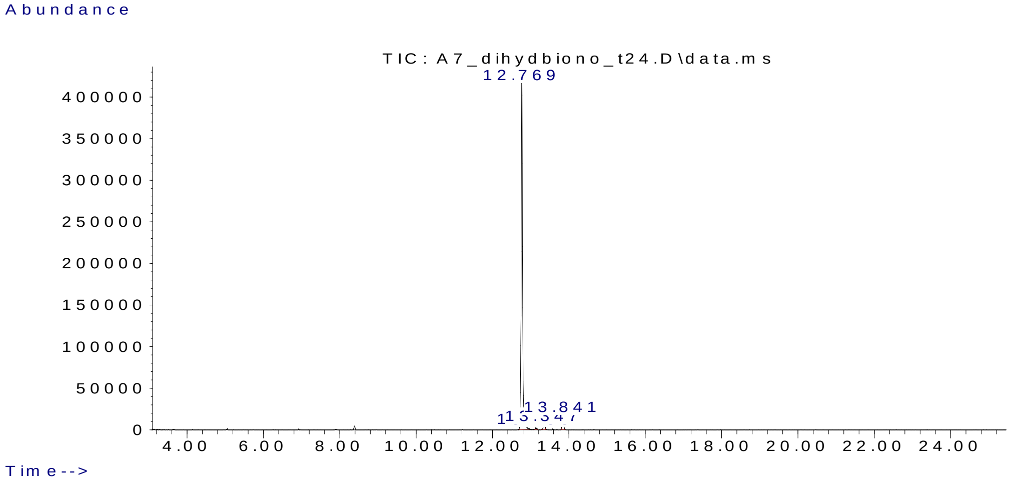

Dihydro-β-ionone **5** (*m/z* = 194): 8.398 min

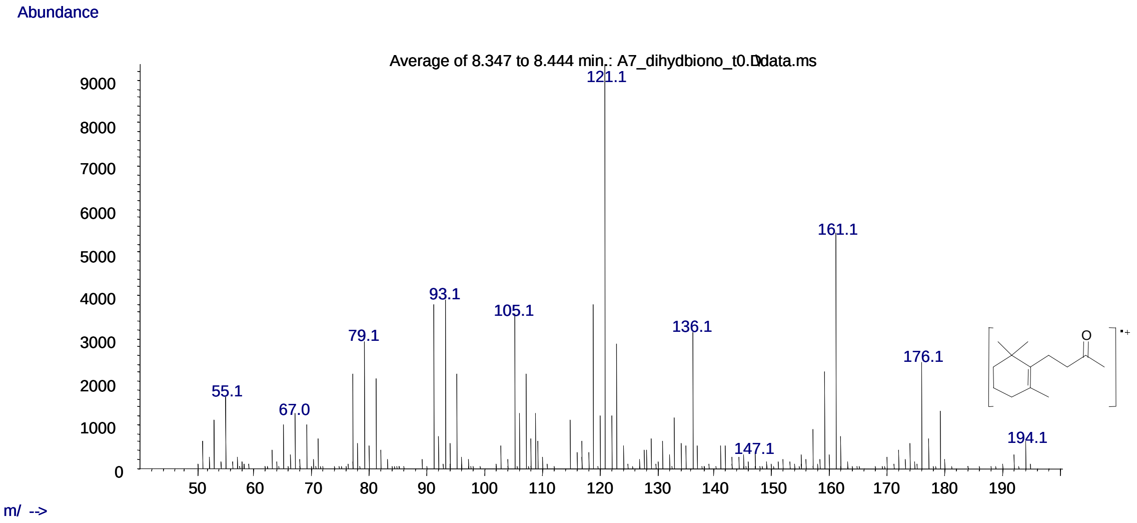

7,8-Dihydro-4-hydroxy-β-ionone (m/z = 210): 12.769 min. Assignment of hydroxylation regioselectivity was based on NMR studies and by comparison with literature MS-data [7].

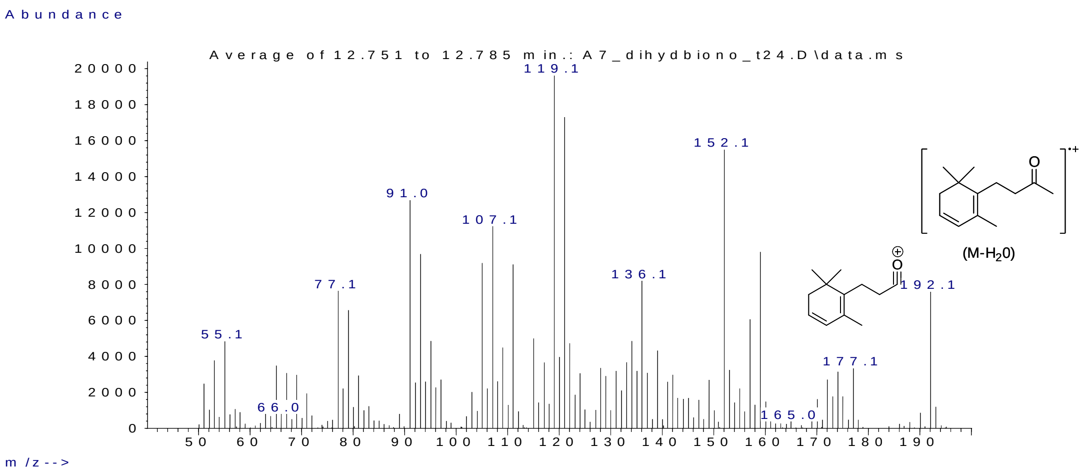

*Biotransformation of compound **7b** (1 mM) with P450cam(Y96F/V247A)-RhFRed*

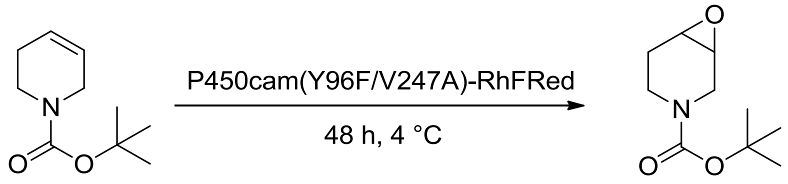

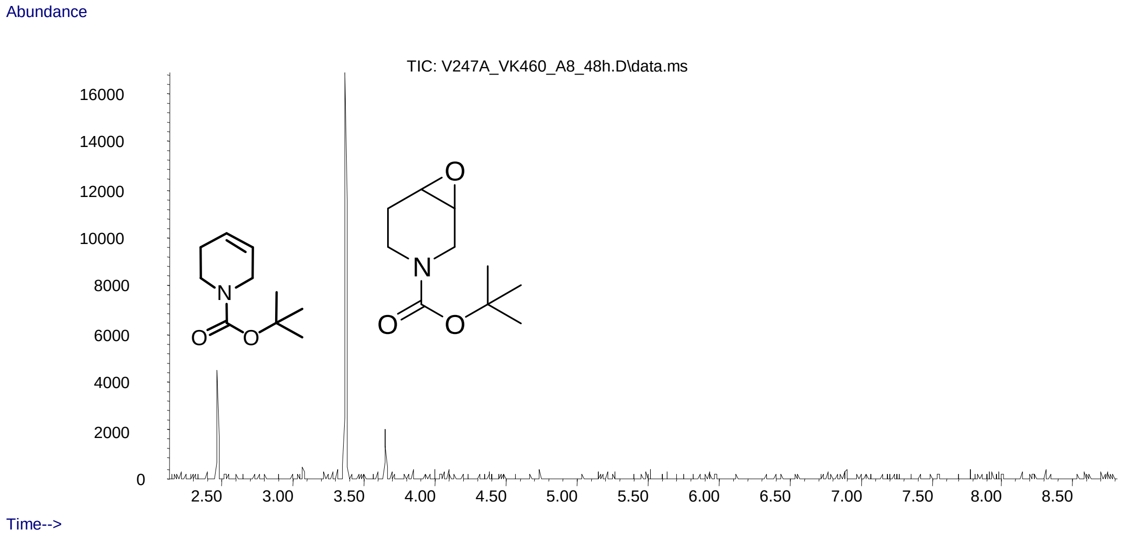

Compound **7b** (m/z = 183): 2.471 min

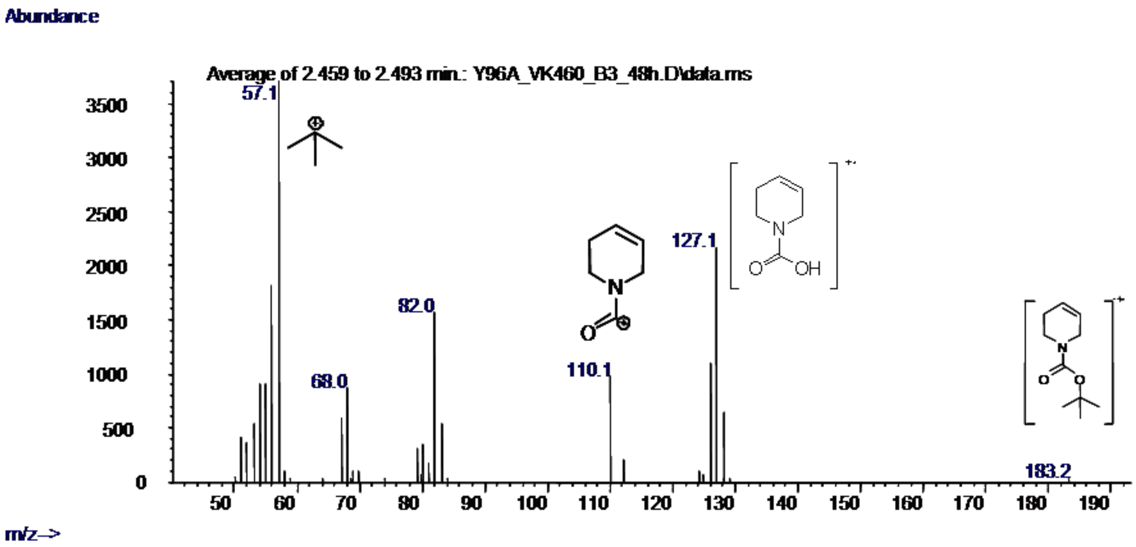

Epoxide product **8a** (*m/z* = 199) : 3.385 min

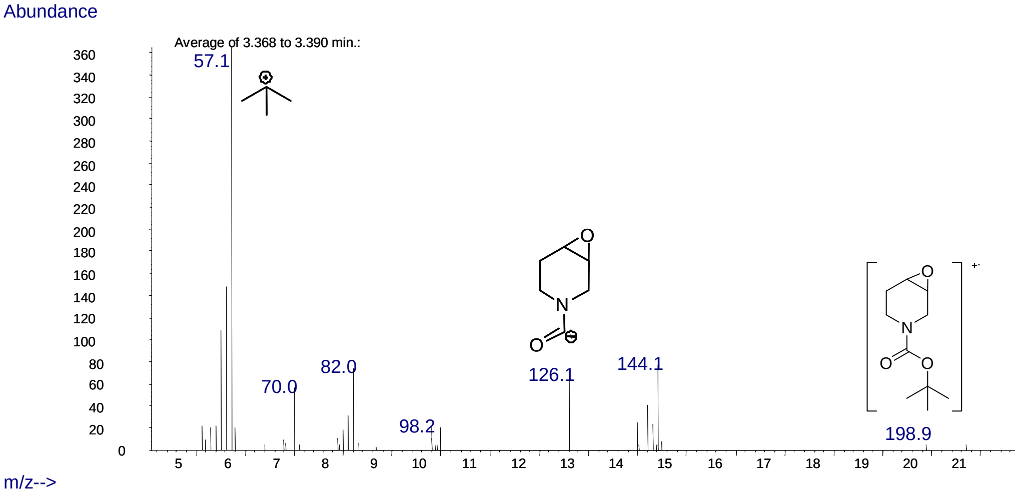

Racemic epoxide product **8a** (synthesised) (*m/z* = 199)

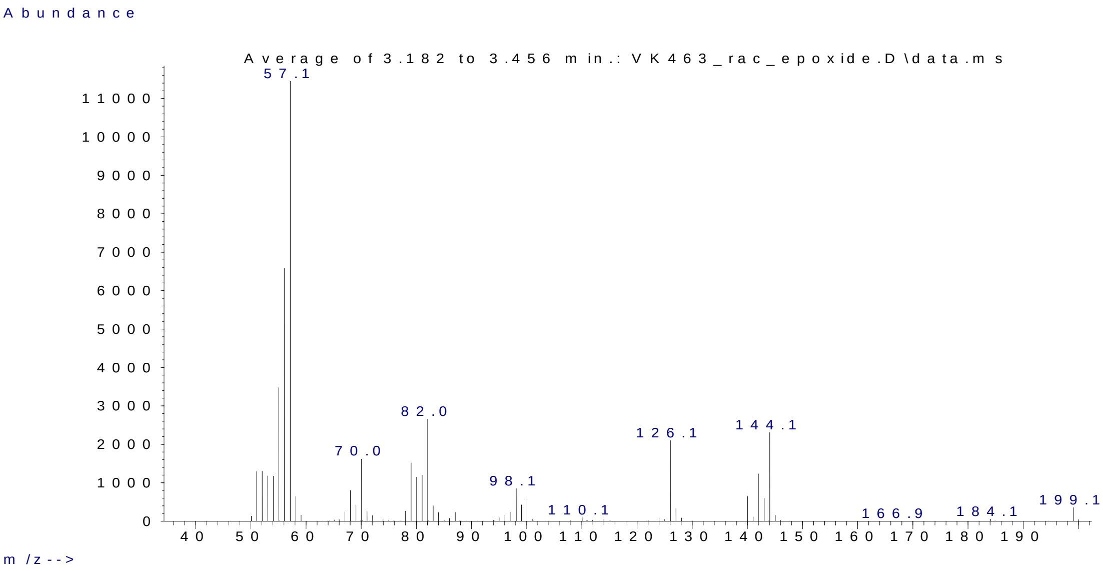

Racemic epoxide product **8a** (synthesised): 13.452 min and 13.999 min (chiral GC-FID).

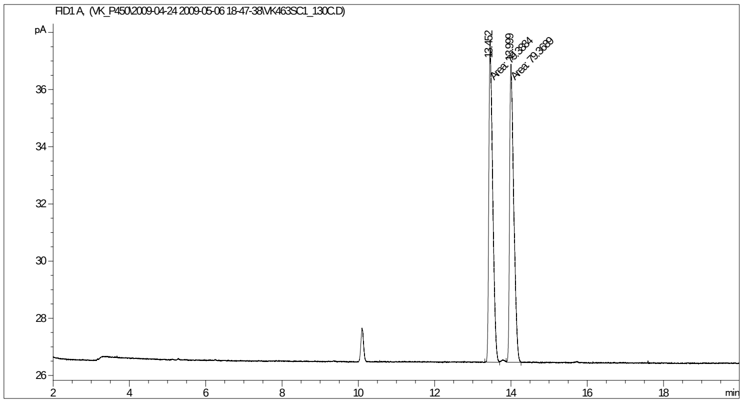

*Biotransformation of compound **7c** (1 mM) with P450cam(Y96F)-RhFRed*

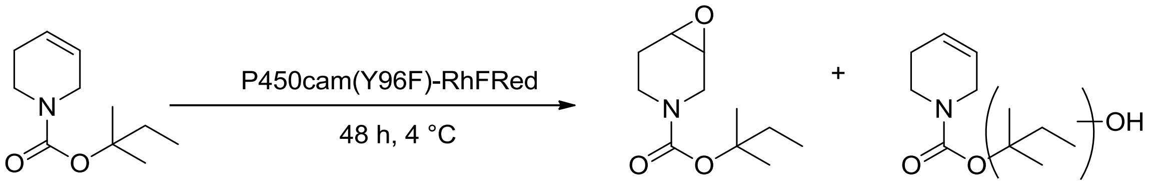

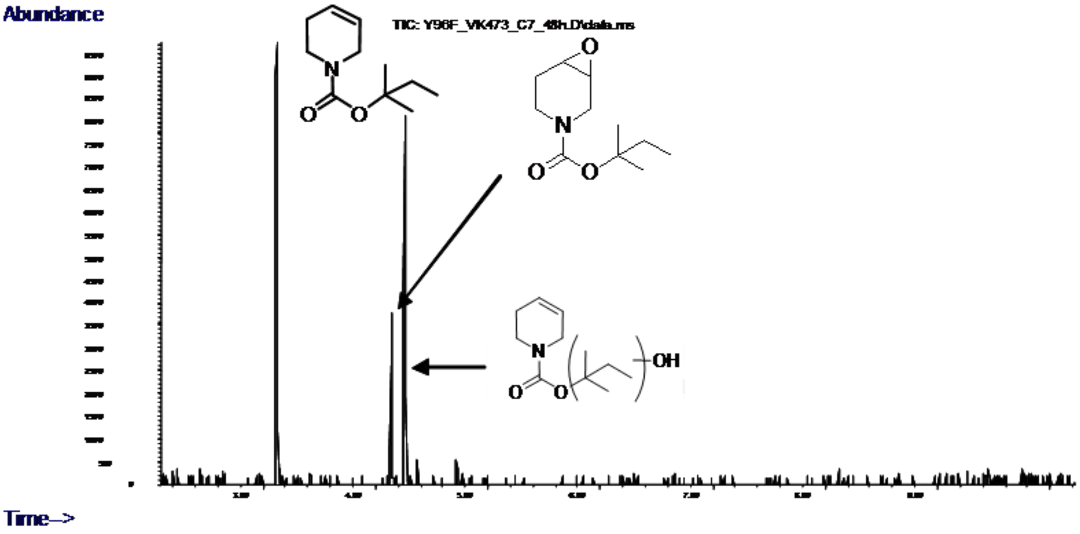

Compound **7c** (*m/z* = 197) : 3.156 min

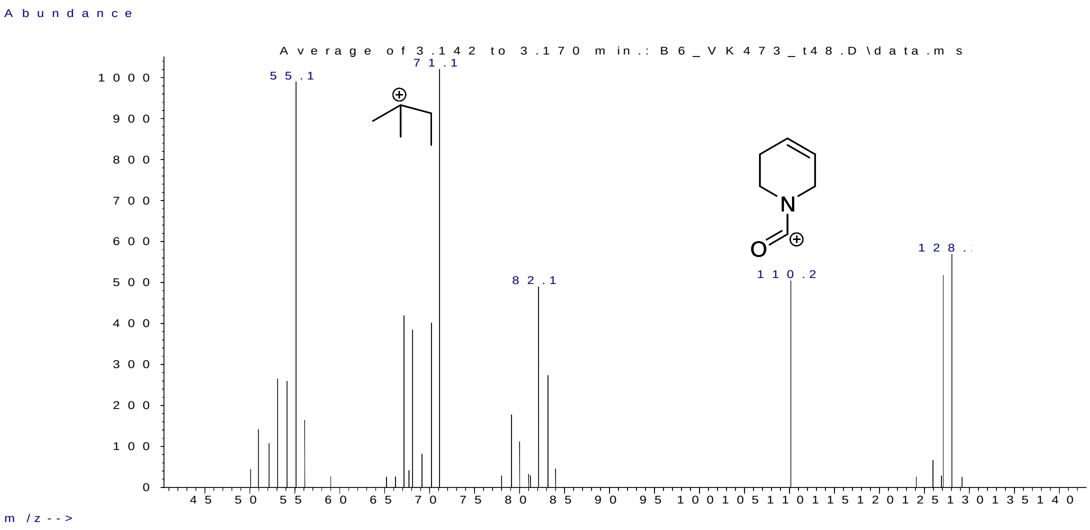

Epoxide product **8b** (*m/z* = 213): 4.142 min

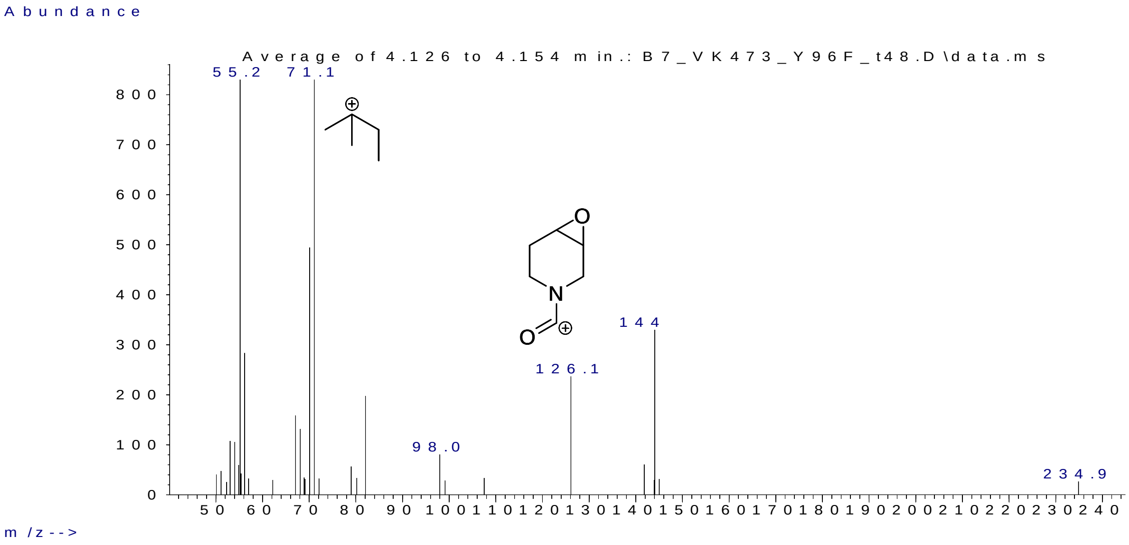

Epoxide product **8b** (synthesised) (*m/z* = 213)

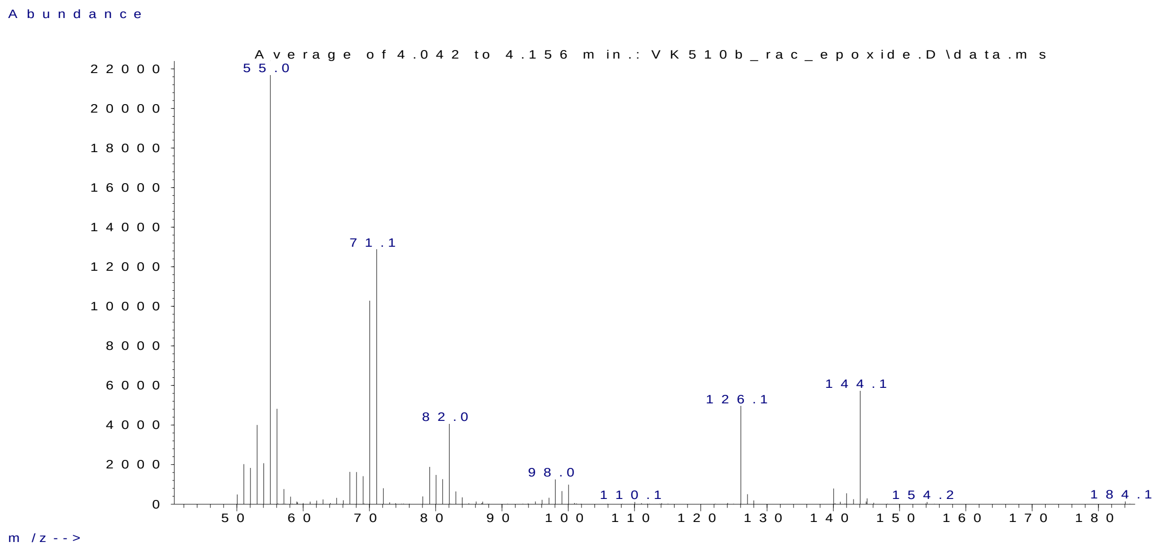

Hydroxylated product **10c** (*m/z* = 213): 4.267 min

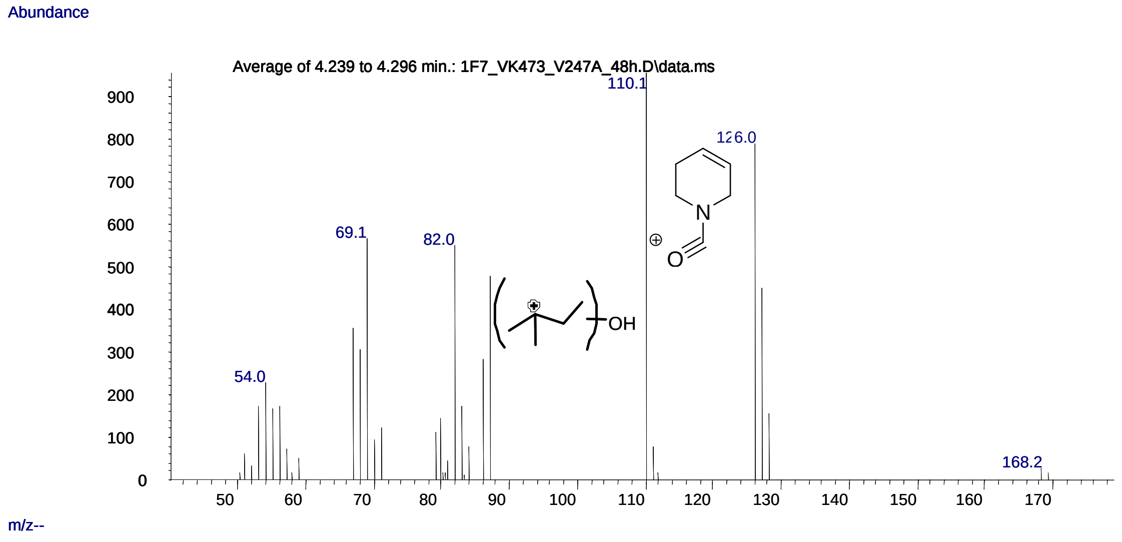

The absence of m/z = 71 and the presence of m/z = 87 indicates the hydroxylation of the protection group.

Biotransformation of compound ***11*** *(1 mM) with P450cam(Y96A)-RhFRed*

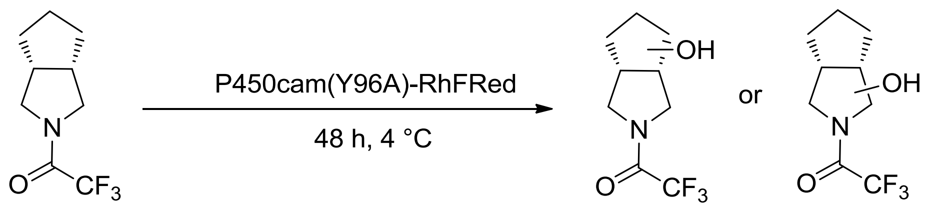

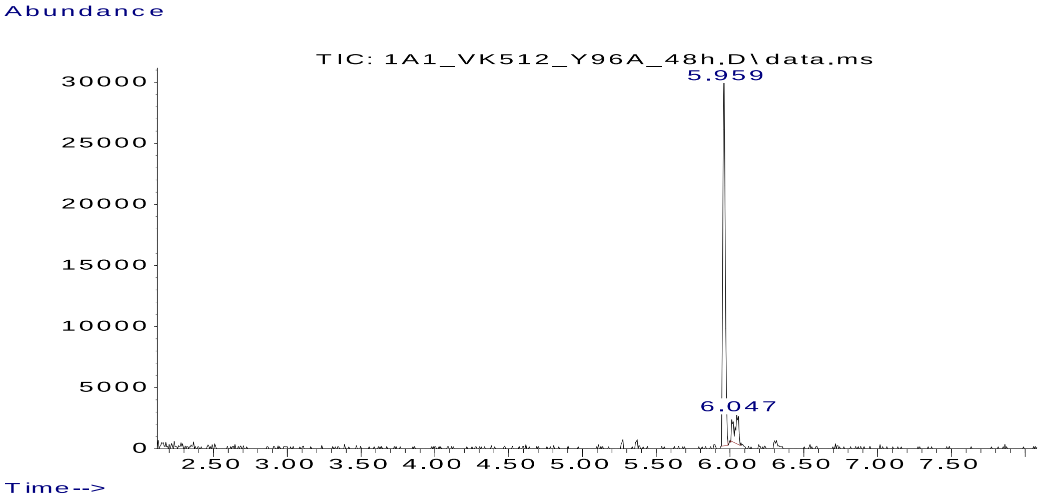

Compound **11** (m/z = 207): 4.358 min

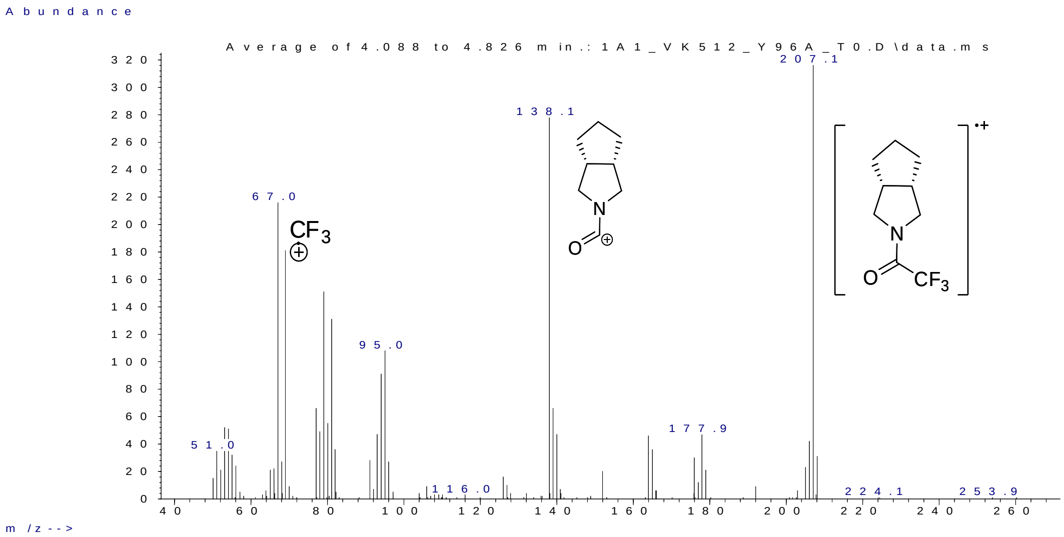

Hydroxylated product **12d** (*m/z* = 223): 5.959 min

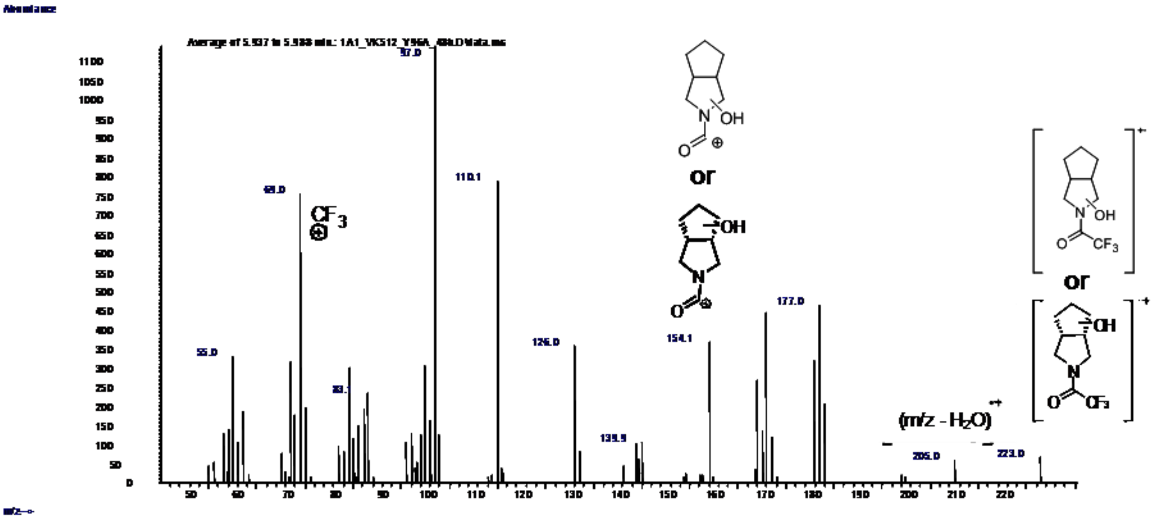

Biotransformation of compound ***13*** *(1 mM) with P450cam(Y96F/V247A)-RhFRed*

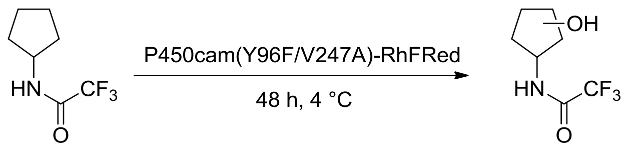

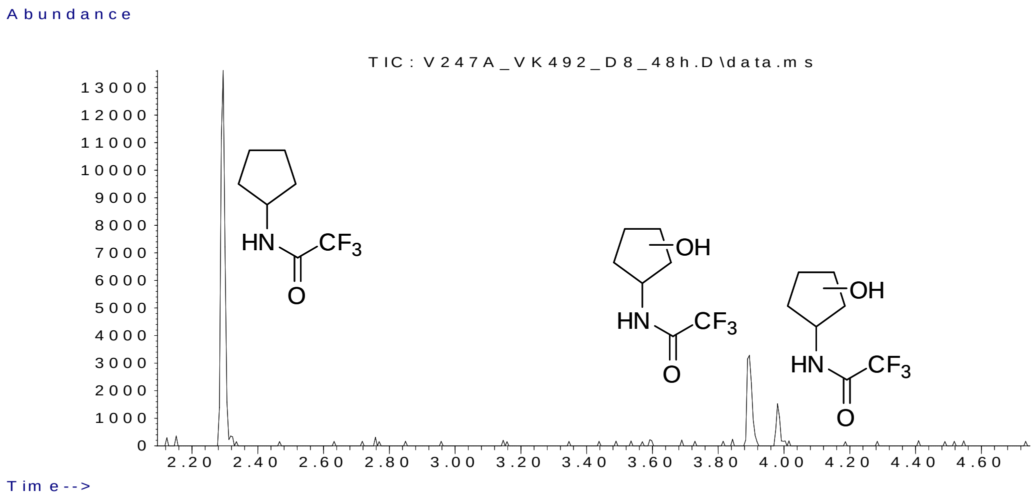

Compound **13** (m/z = 181): 2.296 min

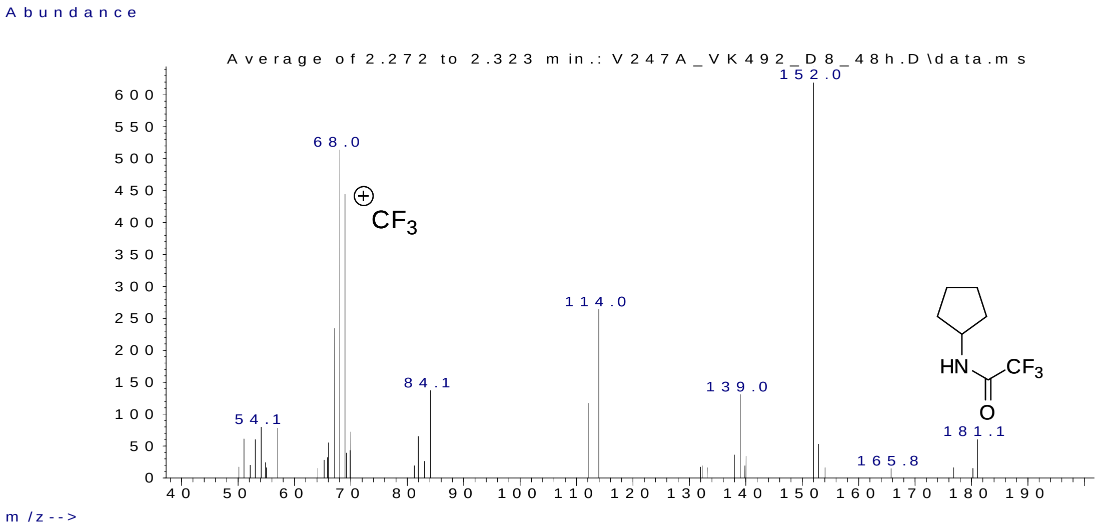

Hydroxylated product **14d** (m/z = 197): 3.893 min

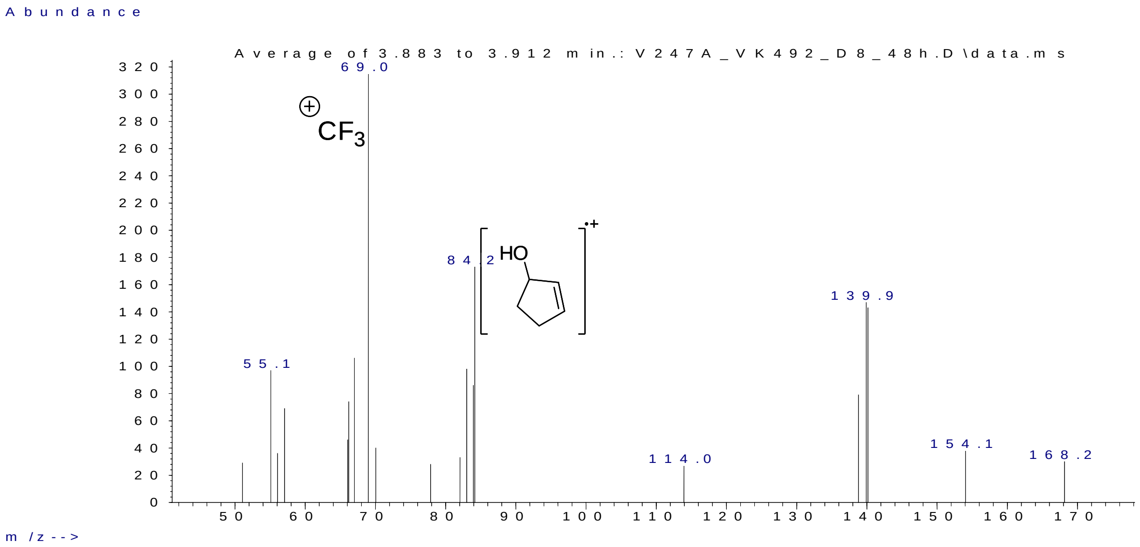

Hydroxylated product **14d’** (*m/z* = 197): 3.981 min

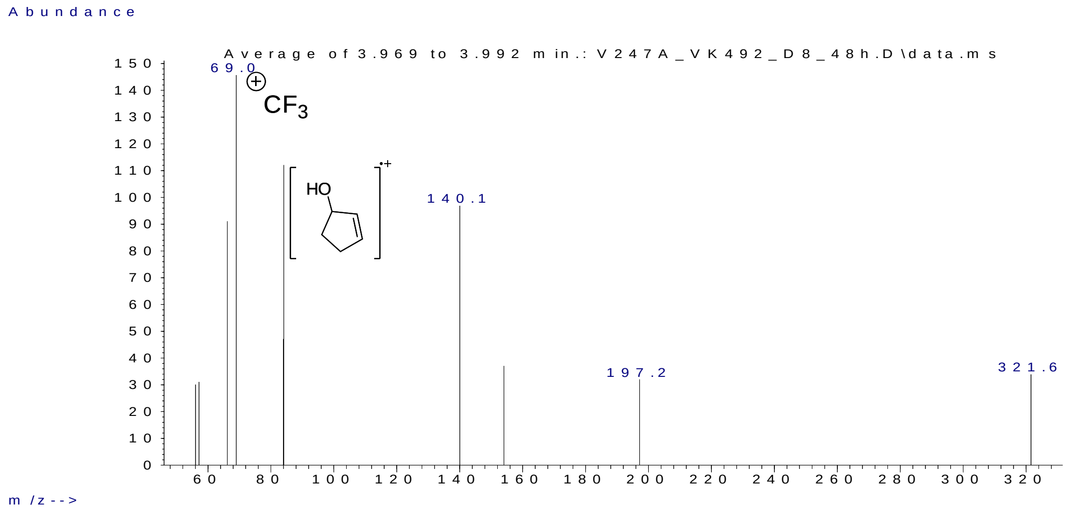

Hydroxylated product **14d** and **14d’** were derivatised to OTMS to confirm the presence of the hydroxyl group (*m/z* = 341):

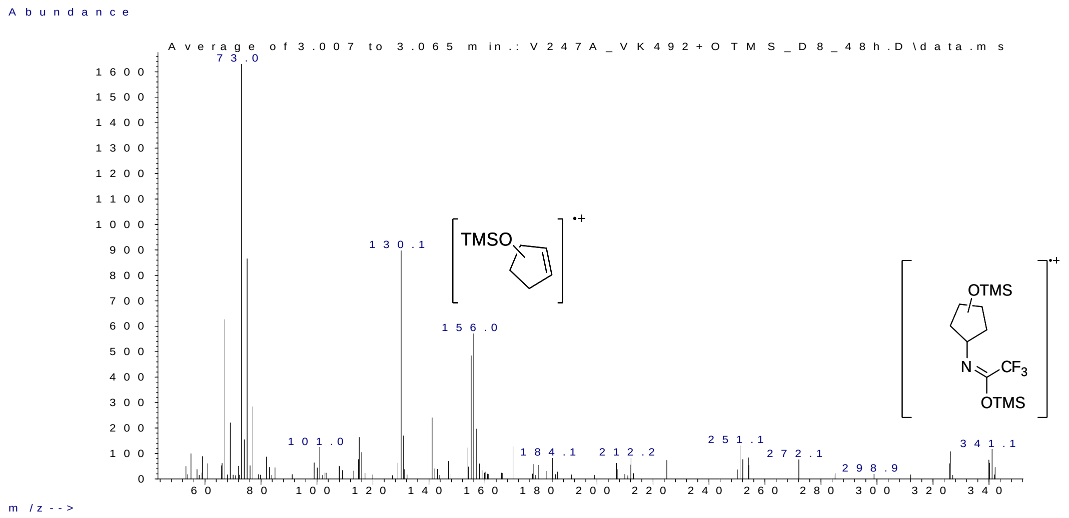

*Biotransformation of compound **15** (1 mM) with P450cam(Y96F/V247A)-RhFRed*

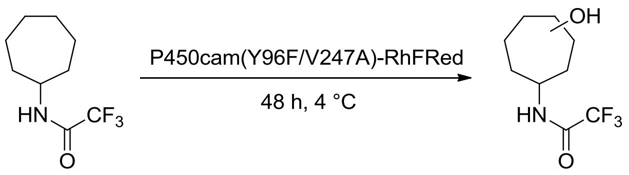

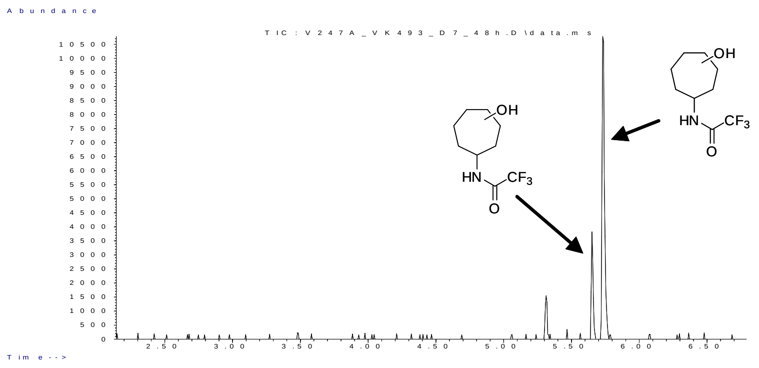

Compound **15** (m/z = 209): 3.902 min

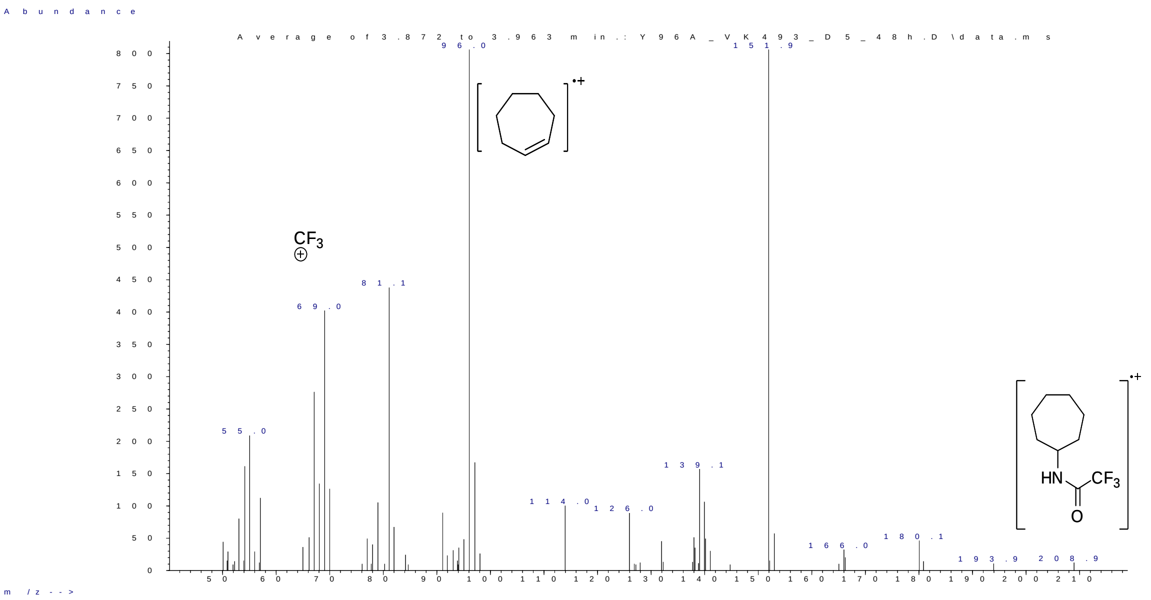

Hydroxylated product **16d** (m/z = 225): 5.654 min

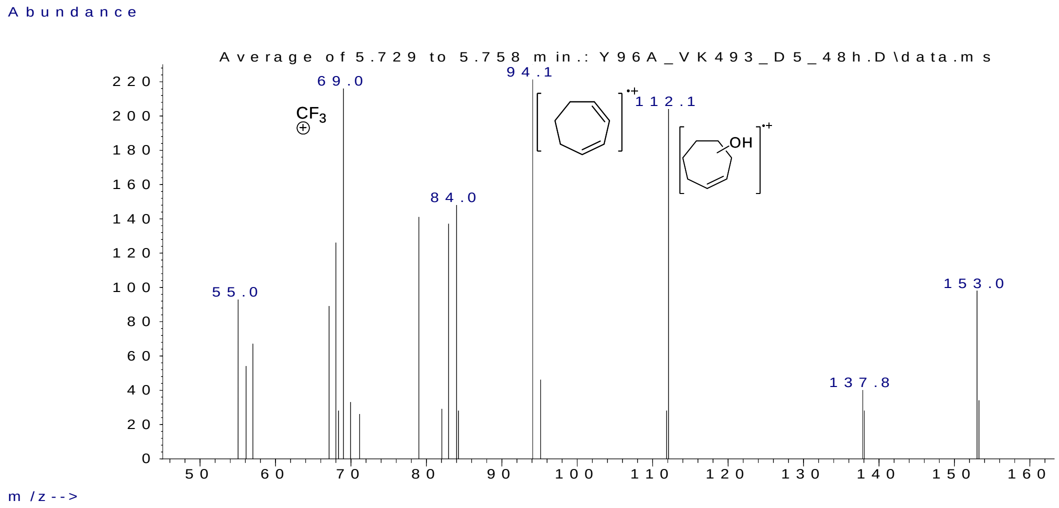

Hydroxylated product **16d’** (m/z = 225): 5.739 min

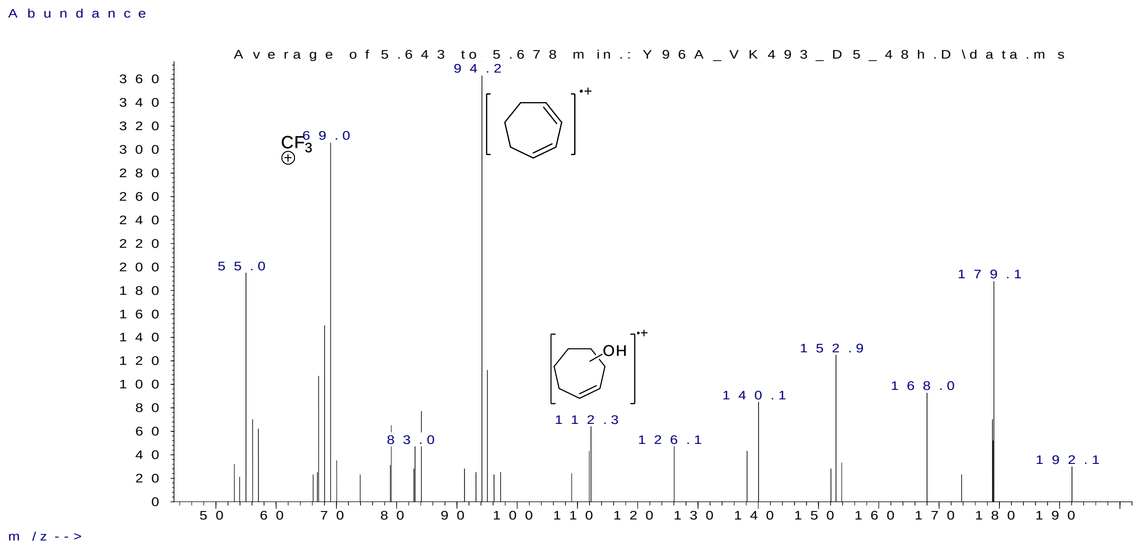

Hydroxylated product **16d** and **16d’** was derivatised to OTMS to confirm the presence of the hydroxyl group (*m/z* = 369):

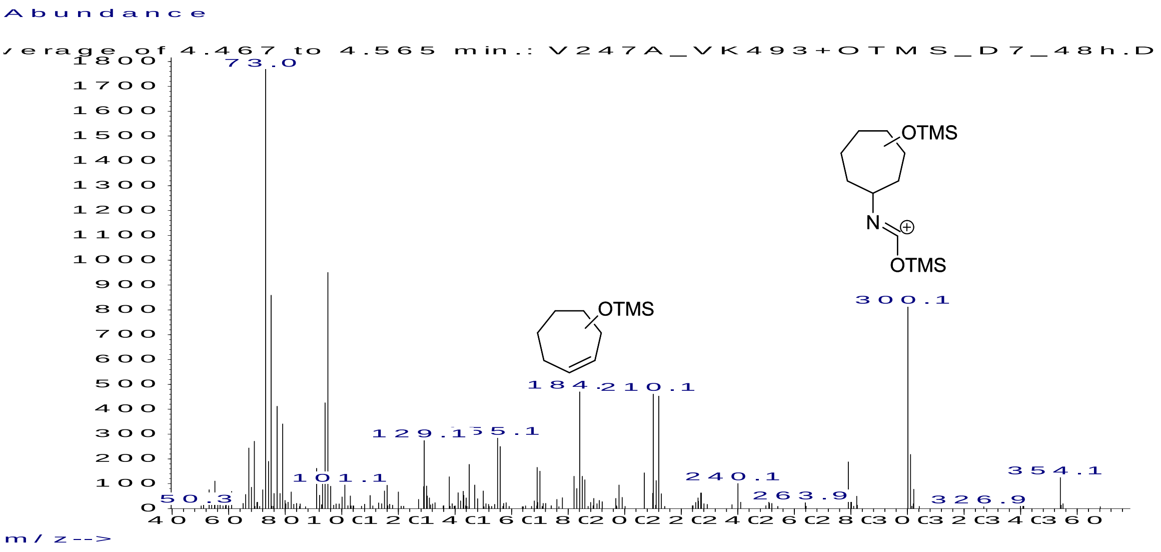

References.

[1] H. Schramm, M. Pavlova, C. Hoenke, J. Christoffers, *Synthesis* **2009**, *10*, 1659-1662.

[2] R. Nordmann, P. Graff, R. Maurer, B. H. Gähwiler, *J. Med. Chem.* **1985**, *28*, 1109-1111.

[3] A. Robin, G. A. Roberts, J. Kisch, F. Sabbadin, G. Grogan, N. Bruce, N. J. Turner, S. L. Flitsch, *Chem. Commun.* **2009**, 2478-2480.

[4] J. Sambrook & D.W. Russell, in *Molecular Cloning: A Laboratory Manual,* Cold Spring Harbor Laboratory Press, Cold Spring Harbor, NY, 3rd edn., **2001**.

[5] A. Celik, S. L. Flitsch, N. J. Turner, *Org. Biomol. Chem.* **2005**, *3*, 2930-2934.

[6] V. B. Urlacher, A. Makhsumkhanov, R. D. Schmid, *Appl. Microbiol. Biotechnol.* **2006**, *70*, 53–59.

[7] A. Pascual, N. Bischofberger, B. Frei, O. Jeger, *Helv. Chim. Acta*, **1988**, *71*, 374-388.
